# Gene expression analysis of induced pluripotent stem cells from aneuploid chromosomal syndromes

**DOI:** 10.1186/1471-2164-14-S5-S8

**Published:** 2013-10-16

**Authors:** Ruosi Zhang, Lili Hao, Lingping Wang, Meili Chen, Wen Li, Rujiao Li, Jun Yu, Jingfa Xiao, Jiayan Wu

**Affiliations:** 1 The CAS Key Laboratory of Genome Sciences and Information, Beijing Institute of Genomics, Chinese Academy of Sciences, Beijing, 100101, China; 2 Graduate University of Chinese Academy of Sciences, Beijing 100049, China; 3 Chinese Academy of Sciences Key Laboratory of Regenerative Biology, South China Institute for Stem Cell Biology and Regenerative Medicine, Guangzhou Institutes of Biomedicine and Health, Guangzhou 510530, China

## Abstract

**Background:**

Human aneuploidy is the leading cause of early pregnancy loss, mental retardation, and multiple congenital anomalies. Due to the high mortality associated with aneuploidy, the pathophysiological mechanisms of aneuploidy syndrome remain largely unknown. Previous studies focused mostly on whether dosage compensation occurs, and the next generation transcriptomics sequencing technology RNA-seq is expected to eventually uncover the mechanisms of gene expression regulation and the related pathological phenotypes in human aneuploidy.

**Results:**

Using next generation transcriptomics sequencing technology RNA-seq, we profiled the transcriptomes of four human aneuploid induced pluripotent stem cell (iPSC) lines generated from monosomy × (Turner syndrome), trisomy 8 (Warkany syndrome 2), trisomy 13 (Patau syndrome), and partial trisomy 11:22 (Emanuel syndrome) as well as two umbilical cord matrix iPSC lines as euploid controls to examine how phenotypic abnormalities develop with aberrant karyotype. A total of 466 M (50-bp) reads were obtained from the six iPSC lines, and over 13,000 mRNAs were identified by gene annotation. Global analysis of gene expression profiles and functional analysis of differentially expressed (DE) genes were implemented. Over 5000 DE genes are determined between aneuploidy and euploid iPSCs respectively while 9 KEGG pathways are overlapped enriched in four aneuploidy samples.

**Conclusions:**

Our results demonstrate that the extra or missing chromosome has extensive effects on the whole transcriptome. Functional analysis of differentially expressed genes reveals that the genes most affected in aneuploid individuals are related to central nervous system development and tumorigenesis.

## Background

Aneuploidy, an abnormal number of chromosomes in humans, is the result of a gain or loss of a chromosome during cell division. Human aneuploidy was first discovered in 1959 by Lejeune and colleagues through monosomy X, also known as Turner syndrome [1]. This is the leading cause of early pregnancy loss, mental retardation, and multiple congenital anomalies [2]. Among first trimester abortions, lethality due to aneuploidy is greater than that from all other causes combined [3]. Scientists have always been interested in determining how aneuploidy affects a fetus, and the molecular mechanisms of this condition have been studied for a long time. However, the high mortality rate associated with aneuploidy limits the capability to study aneuploidy syndromes systematically. As a result, most of the published gene expression studies of human aneuploidy involved patients and/or mouse models of Down syndrome [4-11].

In recent years, several aneuploid human embryonic stem cell (ESC) lines have been established as models for studying human aneuploidy syndromes [12-15], which has expanded the scope of aneuploidy research, leading to investigations of other syndromes caused by the gain or loss of a chromosome. Compared with ESC models, induced pluripotent stem cell (iPSC) models, the successful reprogramming of differentiated human somatic cells into a pluripotent state, can be applied to more easily study human disease [16]. Recently, two laboratories generated iPSCs from patients with an aneuploid syndrome [17,18]. iPSCs were shown to stably maintain the karyotype of the donors and to behave like ESCs [17]. Due to the outstanding performance of the RNA-seq method, analyzing the gene expression profiles of these aneuploid iPSCs will provide a great way to understand the pathological mechanisms of human aneuploidy.

Several recent studies based on DNA microarray techniques concluded that an extra or missing chromosome may have a major effect on gene expression on the particular chromosome but only a minor effect on the whole transcriptome [4,8,9,19]. Conversely, some other studies suggested the extra or missing chromosome has a global effect on the whole transcriptome that is regulated by dosage compensation [20,21]. Dosage compensation is a process that mainly restores gene dosage to a balanced level between × chromosome and autosomes in mammals and has been reported in an aneuploid condition [22]. With the influence of dosage compensation, some genes on the extra or missing chromosome will have no change in gene product levels compared with disomic controls [23]. However, Xiong and colleagues found there is no dosage compensation of the active × chromosome and revised the current model of dosage compensation with RNA sequencing, revealing that with application of next generation sequencing technologies, the mechanism of gene expression regulation and its related pathological phenotypes in human aneuploidy eventually can be discovered [24-26].

To examine how phenotypic abnormalities develop with aberrant karyotype, we profiled the transcriptomes of four human iPSC lines by RNA-seq technology on a next generation sequencing platform. The four iPSC lines were generated from monosomy × (Turner syndrome), trisomy 8 (Warkany syndrome 2), trisomy 13 (Patau syndrome), and partial trisomy 11:22 (Emanuel syndrome), which are seldom associated with postnatal survival. We compared the gene expression profiles of the four aneuploid iPSCs with those of two iPSCs generated from umbilical cord matrix cells (UMCs) as euploid controls and attempted to discover how the extra or missing chromosome affects the human transcriptome and the specific transcriptional changes caused by dosage imbalance. Functional analysis of differentially expressed (DE) genes allowed us to determine the significance of several processes in aneuploidy during embryonic development. The aim of this study was to explain how aneuploidy disrupts fetal development and contributes to phenotypic variations in order to better understand the molecular etiopathology of aneuploidy.

## Results

### SOLiD transcriptome sequencing of aneuploid and euploid iPSCs

We generated a highly detailed transcriptome profile for four aneuploid iPSC and two euploid iPSC clones using RNA-seq. The creation of iPSCs from UMCs is easy to achieve and produces large numbers of cells that escape acquired somatic cell mutations, which were applied as euploid controls (UMC1 and UMC6). All transcriptome libraries were generated and sequenced on a SOLiD v3 platform (Applied Biosystems, Foster City, CA, USA). We obtained 59.5 M and 58.2 M (50-bp) reads from the two UMC samples and 83.1 ~ 90.7 M (50-bp) reads from the four aneuploid samples.

Sequenced reads were mapped onto the human genome (hg19) using Corona Lite (See Methods, detailed mapping results are given in Table 1A). Approximately 41-47% of reads from the four aneuploid iPSC lines were uniquely mapped onto the reference genome, compared to only 24% and 33% reads uniquely mapped in euploid controls. Only the uniquely mapped reads were used for further analysis, most of which (66-77%) were mapped onto exons. To examine the influence of this mapping discrepancy, we conducted a saturation experiment. As shown in Additional File 1, the data set with the fewest mapped reads, UMC1, had a saturation curve fairly close to the horizontal line. Thus, the transcriptome sequencing was deep enough and the discrepancy between samples can be eliminated after normalization.

**Table 1:** Statistical information of RNA-seq mapping result.

A. Number of reads in each cell line						
**Sample**	**Total reads**	**Total Mapped**	**Percent**	**Unique Mapped**	**Percent**	

UMC1	59,478,926	19,119,147	32.14%	14,256,083	23.97%	
UMC6	58,161,509	26,559,086	45.66%	19,416,912	33.38%	
T8	86,659,524	46,672,781	53.86%	36,393,943	42.00%	
T13	90,676,957	49,763,484	54.88%	38,656,136	42.63%	
T22	83,120,658	49,582,084	59.65%	38,498,635	46.32%	
XO	88,052,908	46,906,900	53.27%	36,512,405	41.47%	

B. Gene expression levels. More than 80% genes are moderately expressed.						

**Expression Level (RPKM)**	**UMC1**	**UMC6**	**T8**	**T13**	**T22**	**XO**

Low 0.3-1	1755	1762	1817	1831	1933	1850
Medium 1-100	11350	11270	11548	11534	11041	11060
High > 100	299	331	281	255	250	295
Total	13404	13363	13646	13620	13224	13205

A. Number of reads (total reads, mapped reads and unique mapped reads) shows in every cell lines. B. Distribution of all expressed genes among different expression levels. More than 80% genes are medium expressed.

**Figure 1:**
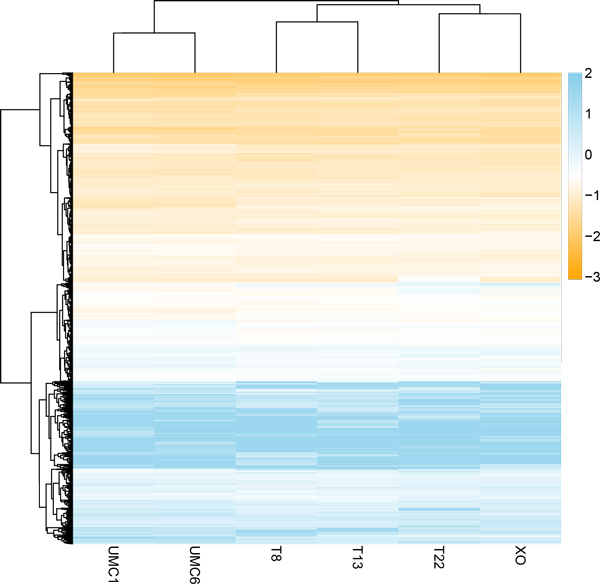
**Hierarchical clustering results of gene expression data**. Four aneuploid iPSC lines are trisomy 8 (T8), trisomy 13 (T13), partial trisomy 11:22 (T22), and monosomy × (XO). Two euploid iPSC lines are UMC1 and UMC6. Columns represent cell lines and rows represent genes. Fold change values compared to mock are represented using log2 expression according to the color key on the right.

**Figure 2:**
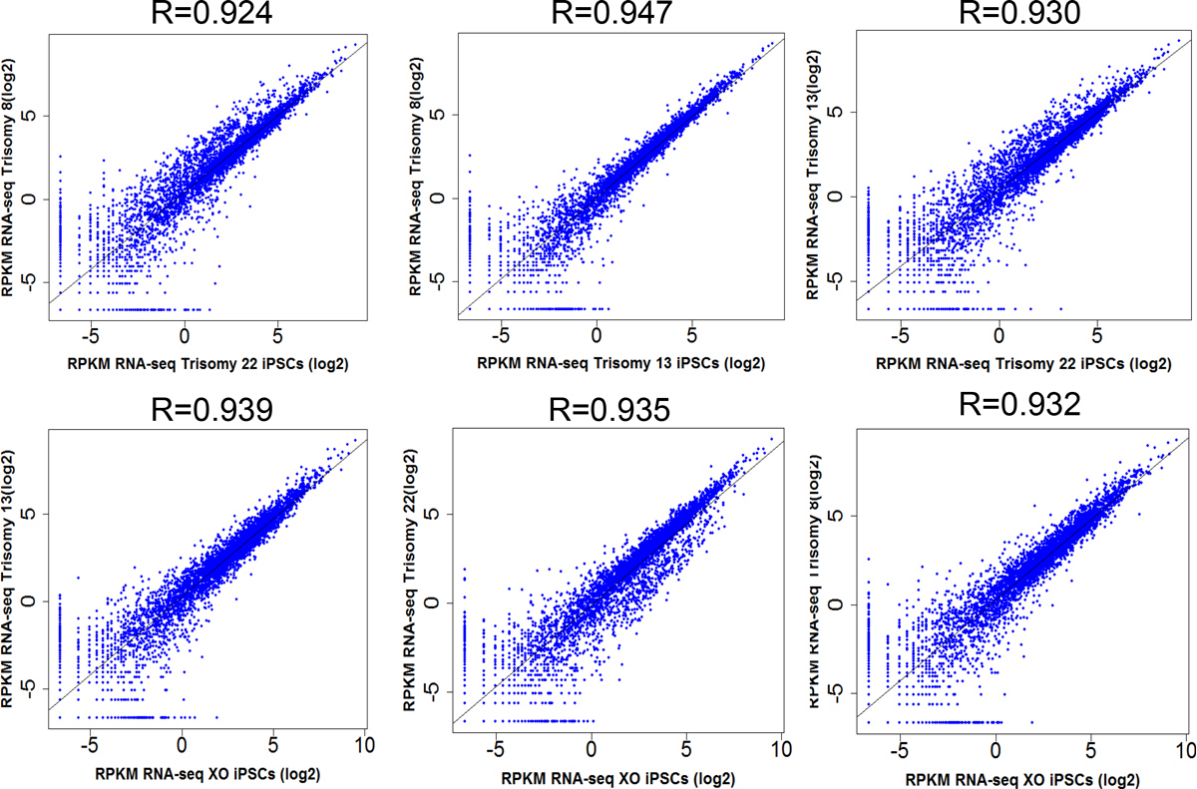
**Pearson's correlation coefficient scatter plots between all aneuploid iPSCs**.

Read densities for each gene were calculated by the number of uniquely mapped reads per kb per million mapped reads (RPKM), and over 13,000 mRNAs were identified by gene annotation (Table 1B). Hierarchical clustering of gene expression data showed that aneuploid samples exhibit similar expression profiles (Figure 1), whereas euploid iPSC clones generated from UMC were most similar to each other. The expression differences between aneuploid and euploid iPSC clones were minor on a global scale, which agrees with the published microarray data showing that Turner syndrome iPSCs exhibited clustering isolated from normal iPSCs with minor discrepancies [17]. To further investigate the expression differences between aneuploid and euploid iPSCs, we calculated Pearson's correlation coefficients between the six cell lines. The scatter plots between all aneuploid iPSCs are presented in Figure 2, and the scatter plots of UMC1 and UMC6 are presented in Additional File 2. The correlation analysis showed that the expression differences at the whole transcriptome level are not significantly different between aneuploid and euploid clones (Table 2).

**Table 2:** Pearson's correlation coefficients between all aneuploid and euploid iPSCs.

	UMC1	UMC6	T8	T13	T22	XO
UMC1	1	-	-	-	-	-
UMC6	0.967	1	-	-	-	-
T8	0.939	0.942	1	-	-	-
T13	0.924	0.942	0.947	1	-	-
T22	0.939	0.925	0.924	0.930	1	-
XO	0.931	0.935	0.932	0.939	0.935	1

### Differential gene expression between aneuploid and euploid iPSCs

**Figure 3:**
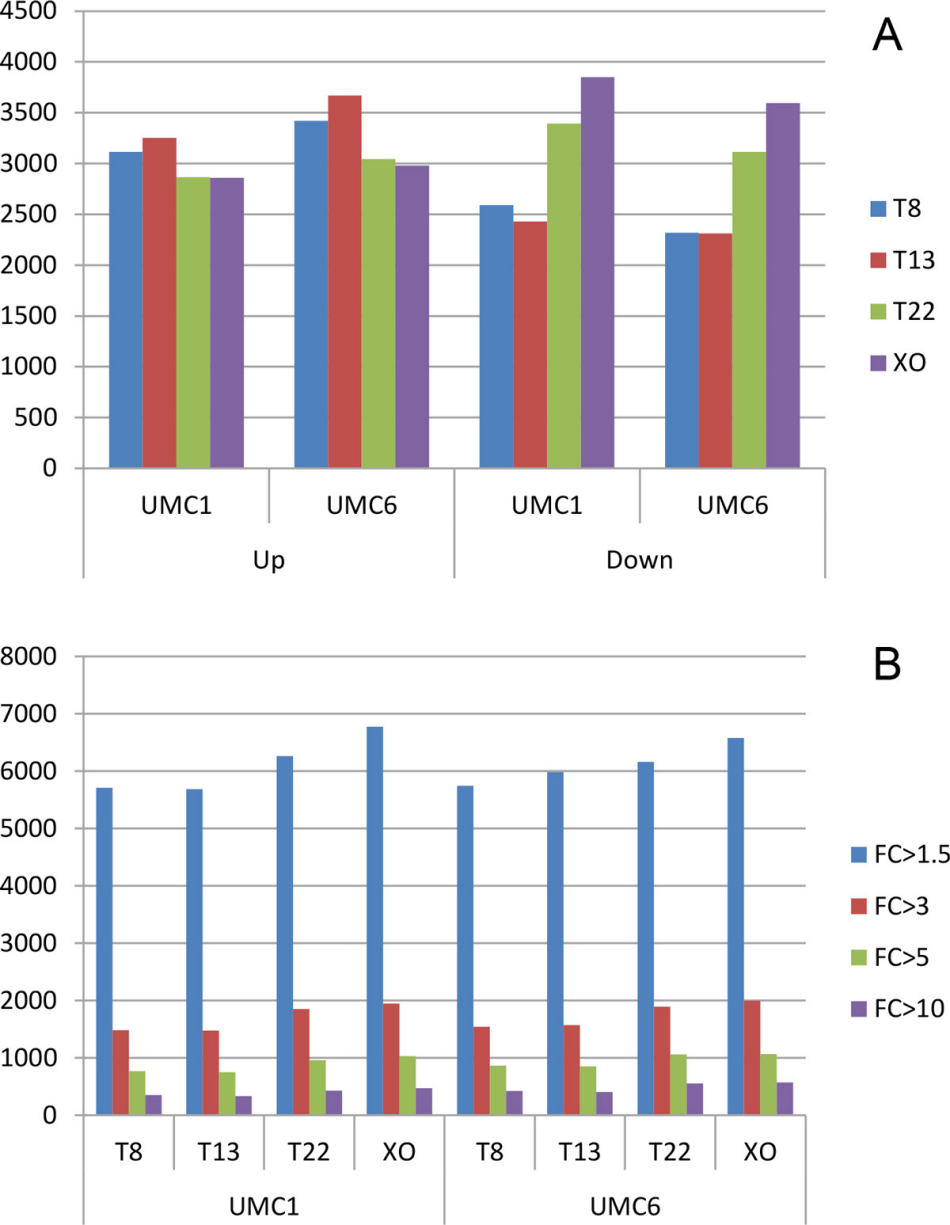
**DE genes amount between euploid and aneuploid iPSCs**. The threshold is P value≤0.05, and Q value≤0.05. A. DE genes are classified into up- or down-regulated genes with fold change>1.5. B. DE genes are calculated with different cut-offs of fold change (FC).

We considered a gene to be significantly DE between two iPSC lines if P-values and Q-values of DEGseq results were both less than 0.05. If one gene is both up-regulated or both down-regulated in UMC1 and UMC6 compared to one aneuploid cell line, it is classified as a "both" up-regulated or down-regulated gene. We find that more than 60% of up- or down-regulated genes in aneuploid clones are "both" up- or down-regulated genes, confirming the differences between aneuploid and euploid iPSC clones. The numbers of up- and down-regulated genes in each aneuploid line were generally similar. There were more up-regulated genes in trisomy 8 and trisomy 13, whereas there were more down-regulated genes in trisomy 22 and monosomy × (Figure 3A, Additional File 3). Compared to previous transcriptome analysis of trisomy 13 and trisomy 8 with DNA microarray [19,20], RNA-seq data detects more signal of expressed genes. Thus, microarray results may not be able to reliably identify differential expressed genes with small fold change [27], while RNA-seq technology perform excellently in measuring gene expression levels with enough depth and sensitivity [28].

**Figure 4:**
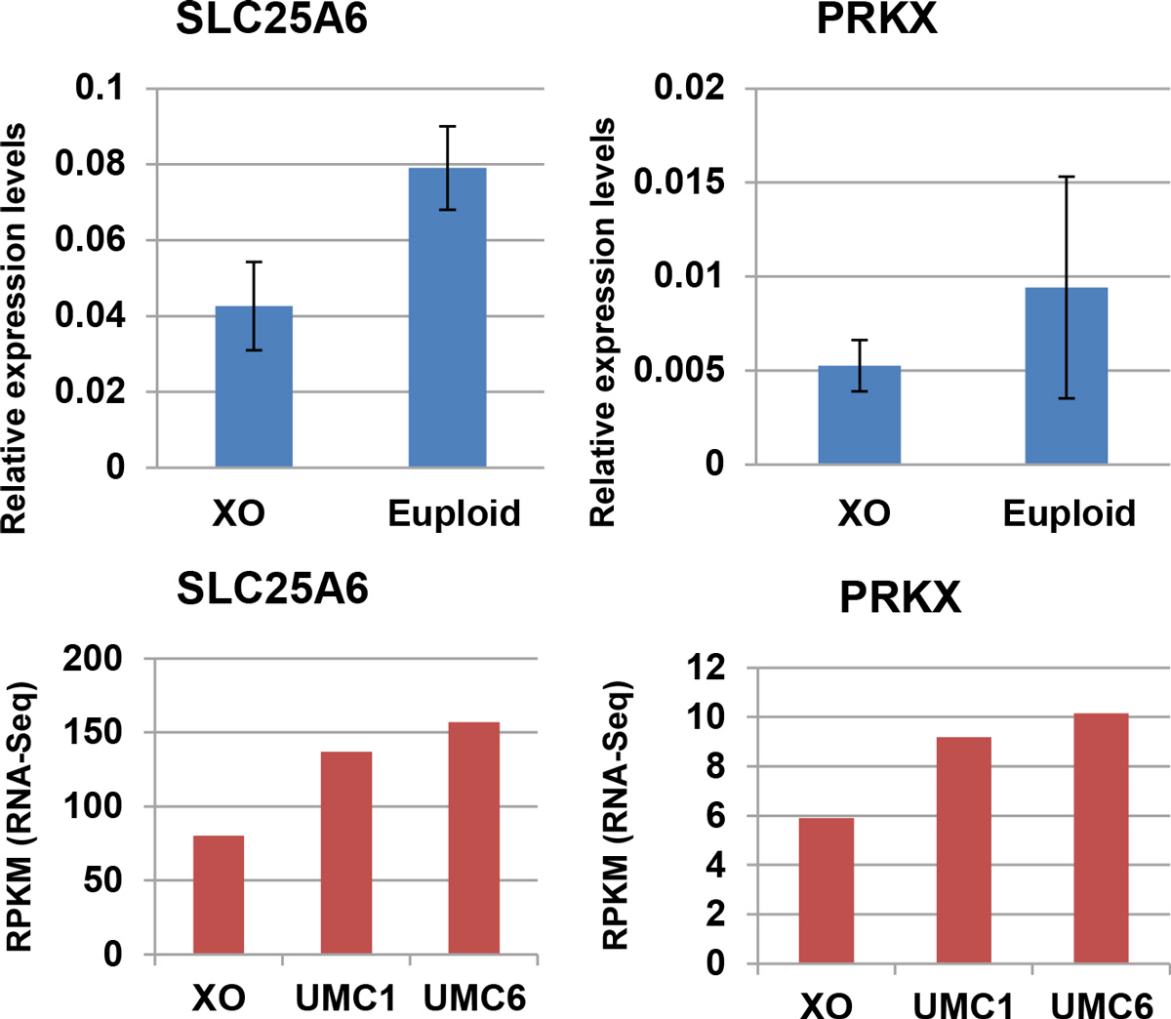
**Validation of RNA-seq results by qPCR**. SLC25A6 and PRKX are selected and performed qPCR experiments for the validation of mRNA expression. The relative expression values of SLC25A6 and PRKX are nearly 2 fold in euploid sample than in XO. Values are referred to the respective iPS cell lines.

We used a more stringent fold change cut-off value to define DE genes between aneuploid and euploid iPSC clones (Figure 3B) and found that the number of DE genes between aneuploid and euploid clones was decreasing dramatically. With a fold-change cut-off of 1.5, 26-34% of expressed genes were DE, which decreases to only 6-8% with a fold-change cut-off of 3 and falls even further to 3-4% with a fold-change cut-off of 5. These results confirm that aneuploidy has a dosage effect on gene expression levels. We selected two genes, *SLC25A6 *(Solute Carrier Family 25, Member 6) and *PRKX *(protein kinase X), to validate our RNA-seq results by quantitative PCR (qPCR) in XO cell line and euploid cell line. The relative expression levels of both genes are nearly 2 fold in euploid sample than in XO, which is in accordance with the differential level of gene expression by RNA sequencing (Figure 4).

**Figure 5:**
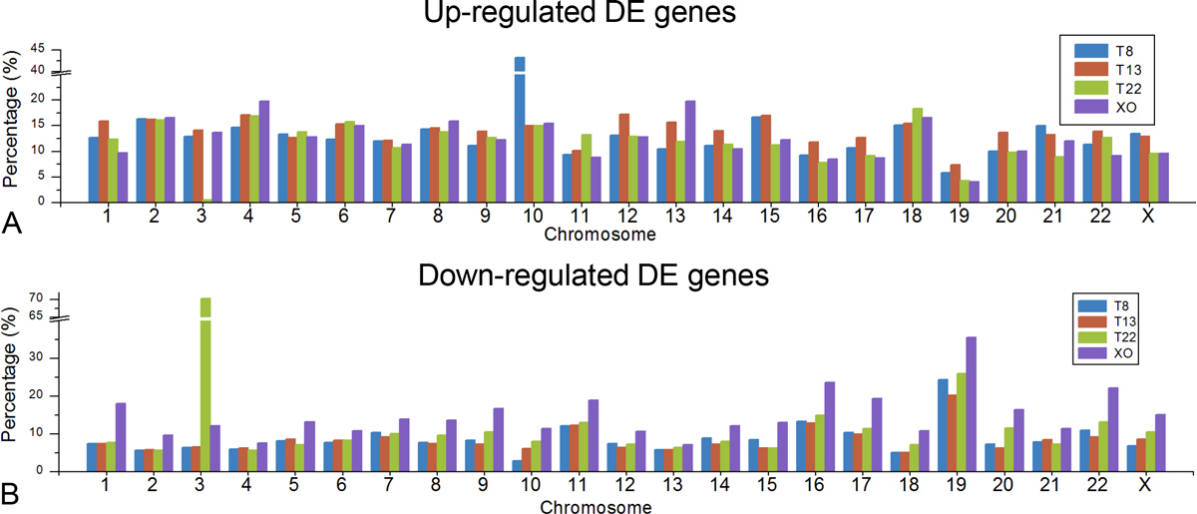
**Gene regulation distribution on each chromosome**. Percentages of DE genes out of all expressed genes on each chromosome are shown as up-regulated part (A) and down-regulated part (B). On y-axis, breaks in scale are introduced because of the high percentage of chromosome 10 in T8 and chromosome 3 in T22.

We examined the up or down regulation of all expressed genes on each chromosome, and we found that transcriptome regulation is ubiquitous on all chromosomes not just on the extra chromosome or single remaining chromosome (Figure 5). In the four aneuploid cell lines, 8-20% of genes on each chromosome were up regulated, whereas the percentage of down-regulated genes varied between 5% and 24%, a slightly wider range than for up regulation. The exceptions were chromosome 19 in all four aneuploid lines, chromosome 3 in trisomy 22, and chromosome 10 in trisomy 8. The exceptional performance of gene expression regulation on chromosome 19 was very similar among the four aneuploid samples, with less than 10% of genes up-regulated and more than 20% down-regulated (as high as 35% in monosomy X). For chromosome 3 in trisomy 22, a very low percentage (only 0.7%) of genes were up regulated, whereas more than 70% of genes on the same chromosome were down regulated. A similar situation occurred on chromosome 10 in trisomy 8, with only 2.9% genes down regulated and 43.3% up regulated. Notably, the ratio of down-regulated genes on each chromosome of monosomy × is much higher than those on other three aneuploid cell lines, which may be caused by the loss of an × chromosome.

### Functional profiling of DE genes

**Figure 6:**
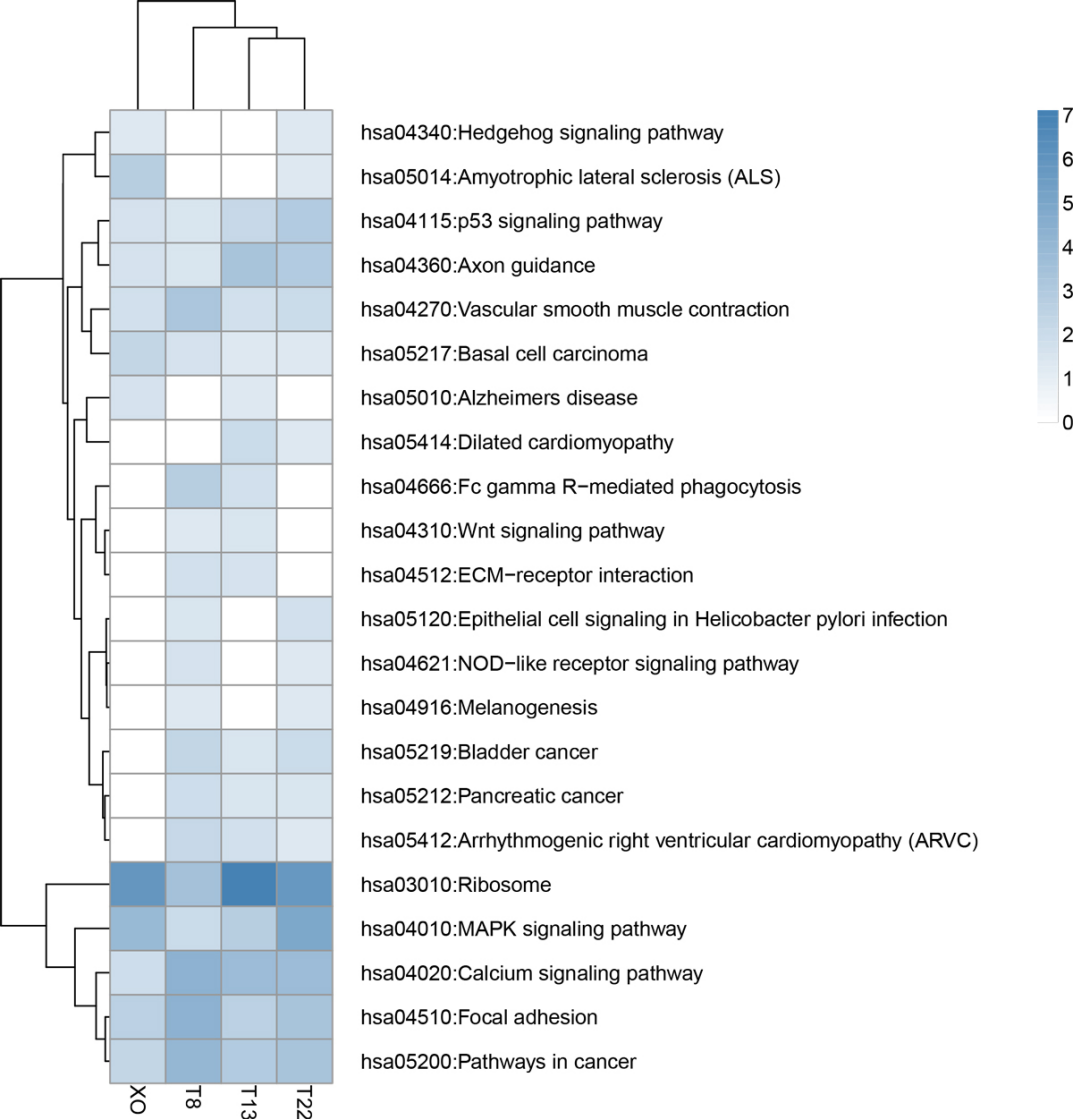
**Clustered heatmap of KEGG pathway enrichment analysis**. Pathways found in more than 2 aneuploid cell lines are shown. The color intensities indicate enrichment score of each KEGG pathway.

The presence of an extra chromosome or absence of a missing chromosome has various molecular effects on aneuploid individuals during fetal development. In order to explore the connection between the functional categories of DE genes and the symptoms of aneuploidy syndromes, we sought to elucidate common regulatory patterns among aneuploid iPSC lines. Functional clustering analysis of DE genes between each aneuploid line and the two euploid controls was performed using Database for Annotation, Visualization, and Integrated Discovery (DAVID) [29]. Following the online instructions provided by DAVID, we examined KEGG pathways and Gene Ontology (GO) terms with P-values less than 0.05 and gene counts more than 2. We identified 28 KEGG pathways for trisomy 8, 19 KEGG pathways for trisomy 13, 23 KEGG pathways for trisomy 22, and 18 KEGG pathways for monosomy X. There are nine pathways appeared in all four aneuploid cell lines: axon guidance, calcium signaling, focal adhesion, ribosome, MAPK signaling pathway, p53 signaling pathway, vascular smooth muscle contraction, pathways in cancer and basal cell carcinoma (Figure 6). GO terms found in all four aneuploid lines are shown in Additional File 4. The biological processes of GO terms in all aneuploid cell lines were related to ion transmission, central nervous system, regulation of apoptosis and cell proliferation which is consistent with those identified KEGG pathways.

Due to the exceptional performance of gene expression regulation on chromosome 3 in trisomy 22 and chromosome 10 in trisomy 8, we performed a functional clustering compared with chromosome 22 in trisomy 22 and chromosome 8 in trisomy 8, using chromosome 1 as a control. We found 8 overlapping KEGG/GO terms between chromosome 3 and chromosome 22, out of 17 terms for chromosome 22 in trisomy 22, which indicates there is a functional connection between the chromosome with abnormally regulated genes, chromosome 3, and the extra chromosome, chromosome 22, in trisomy 22. However, we did not find any overlapping KEGG/GO terms between chromosome 8 and chromosome 10 in trisomy 8, probably because there are only 4 terms in chromosome 8 in trisomy 8. KEGG/GO terms of DE genes on chromosome 3 in trisomy 22 and on chromosome 10 in trisomy 8 are listed in Additional File 5.

In addition, we performed pathway analysis for DE genes in the four aneuploid iPSC lines relative to euploid controls using GeneGo Pathway tool (Additional File 6). Among the common functional groups, pathways related to development appeared to be the predominate group among all functional categories. Cell adhesion, cytoskeleton remodeling, and immune response were also the main functional groups identified in each cell line.

## Discussion

### An extra or missing chromosome has global effects on gene expression

Our study provides a comprehensive understanding of four human aneuploid induced pluripotent stem cell (iPSC) lines and two human euploid iPSC lines by transcriptome profiling with high-throughput next generation sequencing to obtain datasets of differential expression genes. Noteworthy, most published works of aneuploidy gene expression analyses have relied on DNA microarray techniques [8,10,11,13,19], a methodology based on hybridization, with well-known limitations such as worse sensitivity on low expression genes. Here the application of next generation sequencing technologies on quantifying gene expression levels help us to better understand the complexity of aneuploidy gene expression patterns as well as the relationship between gene expression and pathological phenotypes.

To investigate how an extra or missing chromosome affects gene expression in aneuploid cells, Mao and colleagues measured the expression of transcripts in different tissue/cell types of trisomy 21 and found that only chromosome 21 shows significant differential expression relative to euploid controls [8]. Similarly, Hisakatsu and colleagues generated artificial trisomy 8 cells and analyzed the gene expression profiles by microarray data. They found higher average gene expression on the additional chromosome 8 but lower average gene expression levels on all non-trisomic chromosomes [20]. However, David and colleagues presented transcriptome analyses of human fetal cells from pregnancies affected with trisomy 21/13 and trisomy 18 amniocyte cells, and the relative expression levels between chromosomes showed a stable pattern with no significant differences between individual RNA samples in microarray experiments [19]. Due to the relatively high uncertainty of microarray methodology, the discrepancies between these expression patterns may have resulted from the differences of specific operations or the selected tissues. Based on the high quality next generation whole transcript sequencing results in this study, we propose that an extra or missing chromosome has extensive effects on the whole transcriptome. We have measured the gene expression profiles deeply enough in three trisomy and XO iPSC lines to demonstrate that gene expression regulation occurs on every chromosome of each aneuploid sample (Figure 5). The percentage of differentially regulated genes on the aneuploid chromosome was not significantly different from other diploid chromosomes. A possible reason is that DE genes on the extra or missing chromosome influence the gene expression regulation on other chromosomes. Likewise, another recent work that using microarray to estimate gene expression value regulated by artificial aneuploidy indicates that the gain of a single chromosome can indeed result in the up or down regulation of 140-202 genes with only 5-20% of up or down regulated genes located on the extra chromosome [30]. Notably, in each aneuploid cell line, less than one-third of expressed genes on the particular aneuploid chromosome are up- or down-regulated when the fold-change cut-off value is set to 1.5 (Figure 5). To a certain extent, this is credible evidence for dosage compensation. Dosage compensation is commonly observed for sex chromosomes according to previous studies, and it could have a similar influence on the aneuploid chromosome. Otherwise this phenomenon could be attributed to a buffering and feedback mechanism.

### Aneuploidy mainly affects the development of nervous system

We investigated how an extra or missing chromosome leads to molecular effects on aneuploid individuals during fetal development by profiling the whole transcriptomes of iPSCs derived from aneuploidy syndromes. In addition to identifying genes relevant to the aneuploid phenotype, we used functional profiling to identify significantly disrupted biological pathways. Nine KEGG pathways were identified in all four aneuploid iPSC lines: axon guidance, calcium signaling, focal adhesion, ribosome, MAPK signaling pathway, p53 signaling pathway, vascular smooth muscle contraction, pathways in cancer and basal cell carcinoma. The top three are all associated with nervous system development.

Axon guidance is an important process in the development of central nervous system, in which attractive and repulsive guidance cues steer axons in the growth cone along specific pathways [31]. There are several signaling pathways of guidance molecules, such as Slit-Robo and Eph/Ephrin, that are also included in the list of GeneGo pathways (Additional File 6). The Slit-Robo signaling pathway primarily provides important molecular cues for axon guidance during the assembly of the nervous system [32]. Recent research using Robo and Slit gene knockout mice has indicated that the Slit-Robo interaction is an integral factor during genesis of the corpus callosum [33] and the key genes of the Slit-Robo signaling pathway are also expressed in human fetal brain [34]. Agenesis of the corpus callosum was observed in mosaic trisomy 8 according to two case reports [35,36] and could be found in 19% of 63 individuals with trisomy 22 [37]. We believe the absence or hypoplastic state of the corpus callosum in aneuploid syndromes is related to the affected axon guidance due to misregulated genes in the Slit-Robo signaling pathway. On the other hand, Ephrin ligands and their cognate Eph receptors guide axons during neural development and are emerging as key players in synapse formation and plasticity in the central nervous system [38]. The central nervous system anomalies in trisomy 13 have been reported to include partial agenesis of the corpus callosum and neuronal heterotopias in the cerebellum [39], and each aneuploidy shows deficiency in neurodevelopment to a different extent.

The focal adhesion pathway is required for both attractive and repulsive cues to guide axon to their specific targets during development of nervous system [40]. Focal adhesions may have other functions such as cytoskeletal dynamics control but they mainly affect trisomy phenotype by influencing axon guidance pathways. Additionally, Slit-Robo and Eph/Ephrin, the two axon guidance pathways, and the focal adhesion pathway all influence retinal development [41-43], which has been reported to lead to an abnormal phenotype in trisomy syndromes [44,45].

It is not surprising that a large number of misregulated genes are involved in the calcium signaling pathway, as this is the first messenger of signal transduction pathways. Ca^2+ ^signals affect axon guidance by mediating the reversal of neuronal migration induced by slit2 gene or pathway [46]. They also play a key role in regulating the neuronal growth cone while mediating growth and turning responses [47], which might be a minor cause of the corpus callosum agenesis observed in aneuploid syndromes. Calcium signaling pathway also contributes to phenotype of aneuploidy with another identified pathway, vascular smooth muscle contraction, which is directly influenced by calcium concentration [48]. Many cardiovascular are diseases are originating from abnormal function in vascular smooth muscle, especially vascular hypertension. Some patients with Turner syndrome (TS) had been found with a higher cardiovascular morbidity [49,50], especially vascular hypertension. Some patients with Turner syndrome (TS) had been found with a higher cardiovascular morbidity [49,50]. Alzheimer's disease might also arise in aneuploidy syndromes through the alterations of Ca^2+ ^levels to cause disturbances [51].

### Aneuploidy and tumorigenesis

Another set of enriched KEGG pathways in all 4 aneuploidy is related to cancer including P53 signaling, pathways in cancer and basal cell carcinoma. The question of how aneuploidy affect cancer initiation and progression has been studied for over a century [52], though its genetic basis remains unclear. Most cancers contain cells that possess a common characteristic of aneuploidy while abnormal number of chromosomes is essential for tumorigenesis [53].

Trisomy 8 and trisomy 13 has been reported to predispose neoplasms, mainly acute myeloid leukemia (AML), suggesting roles of an extra 8 or 13 chromosome in tumorigenesis [54,55]. It has been proved that trisomy 8 is the most frequent trisomy occurred in AML, which leads to tumor-specific gene-dosage effects such as significantly down-regulated apoptosis-regulating genes [56]. Although there is no explicit association between × chromosome genes and neoplasm, basal cell carcinoma, another enrichment pathway in our study, was diagnosed in a TS patient [57,58]. Several recent investigations showed that TS patients have significantly increased risks of tumor, especially in central nervous system, bladder and urethra [57-59].

Cumulatively, our result together with current evidence suggests that besides multiple developmental abnormalities, aneuploidy associate with alterations in the risk for specific cancers. The extra or missing chromosome disrupts global transcription and promotes tumorigenesis effectively by disturbing cancer related pathways. However, the characteristic of lethality to aneuploidy increase the difficulty to investigate whether the gain or loss of a chromosome contributing to tumorigenesis by down-regulated the expression of tumor suppressor genes and/or up-regulated the expression of oncogenes. Further molecular biological studies are needed to assess, and more clinical reports are needed to prove how aneuploidy affects tumorigenesis.

As to the exceptional performance of more DE genes on chromosome 3 in trisomy 22, 4 KEGG pathways enrich on that chromosome, which are axon guidance, colorectal cancer, glycosaminoglycan degradation and endometrial cancer. Three of them are nervous system and tumorigenesis related pathways. This might explain why the down-regulated genes on chromosome 3 in trisomy 22 are much more than those on other chromosomes.

Our Gene Ontology analysis confirms our KEGG pathway results, especially with respect to nervous system development. The integrated results in this study demonstrate that genes involved in nervous system development and tumorigenesis are most affected pathologically in aneuploid individuals. Our results provide initial indications of possible biological pathways affected by aneuploidy based on deeply transcriptome sequencing. In addition, we also offer a better understanding to of the early etiology of congenital anomalies, which may suggest promote future innovative approaches in health treatment.

## Conclusions

Using next generation transcriptomics sequencing technology RNA-seq, we profiled the transcriptomes of four human aneuploid induced pluripotent stem cell (iPSC) lines generated from monosomy × (Turner syndrome), trisomy 8 (Warkany syndrome 2), trisomy 13 (Patau syndrome), and partial trisomy 11:22 (Emanuel syndrome) as well as two umbilical cord matrix iPSC lines as euploid controls. A total of 466 M (50-bp) reads were obtained from six iPSC lines, and over 13,000 mRNAs were identified by gene annotation. Global analysis of gene expression profiles and functional analysis of differentially expressed (DE) genes were implemented to examine how phenotypic abnormalities develop with aberrant karyotype. Our results demonstrate that the extra or missing chromosome has extensive effects on the whole transcriptome. Functional analysis of differentially expressed genes reveals that the genes most affected in aneuploid individuals are related to central nervous system development and tomorigenesis.

## Methods

### Next-generation transcriptome sequencing and data processing

All human iPSC clones presented here were obtained from the South China Institute for Stem Cell Biology and Regenerative Medicine, Guangzhou Institutes of Biomedicine and Health, and have been described before [17]. Library construction was based on a protocol described previously [60,61]. Total RNA of each line was extracted using TRIzol reagent (Invitrogen) according to the manufacturer's instructions. Poly(A)+ mRNA was isolated from total RNA using Oligotex (QIAGEN). RNA was fragmented with RNase III, preparing for constructing transcriptome libraries of each iPSC cell line. Applied Biosystems SOLiD Whole Transcriptome Analysis Kit (http://solid.appliedbiosystems.com) were applied to perform reversed transcription from 140-200 bp isolated RNA fragments into Single-strand cDNA.

Sequence data were generated using SOLiD3 system (Applied Biosystems) following the manufacturer's instructions. RNA-seq reads were mapped onto the human reference genome (NCBI37/hg19) with Corona_lite_v4.2.2 software (Applied Biosystems), setting the parameters for full-length read mapping (50, 45, 40, 35 bp) with 5, 4, 4, and 3 mismatches. Only reads that uniquely mapped to the genome and reads for genes corresponding to mRNA were chosen for subsequent analysis. Reads density for each gene (shown as RPKM value) was calculated by the number of uniquely mapped. Hierarchical clustering was performed in R using the pheatmap package. Pearson correlation coefficients for each pair of iPSC lines were calculated using the log2 RPKM values via cor function in R.

### Detection of DE genes

DE genes between aneuploid iPSCs (T8, T13, T22, and XO) and normal iPSCs (UMC1 and UMC6) were identified by DEGseq, for which R-packages are available under Bioconductor (http://www.bioconductor.org/packages/2.7). DEGseq is a free R package to detect DE genes between two samples with or without replicates of RNA sequencing data [62]. MA plot-based method (where M is the log ratio of the counts between two experimental conditions for each gene, and A is the two group average of the log concentrations of the gene) with a random sampling method (MARS) was selected. DE genes between 4 aneuploid and 2 euploid samples are calculated respectively. The raw count of each gene was used, and function DEGexp was performed for analysis. A gene was considered to be significantly DE if its P-value and Q-value were both less than 0.05. For each gene, the level of change in expression is stated as a fold-change.

### Functional profiling of DE genes

The Database for Annotation, Visualization, and Integrated Discovery (DAVID) was used to identify KEGG pathways and enriched gene ontology categories of DE genes [29]. Here DE genes between 4 aneuploid samples and UMC1/UMC6 are calculated respectively, then those DE genes expressed in UMC1 or UMC6 are selected to be DAVID input datasets. Following the instructions of DAVID manual, datasets of each sample were uploaded and the function charts were generated. The functional groups with a P-value less than 0.05 and gene counts greater than 2 were examined. Pathway maps of a manually curated proprietary database (MetaCore™, GeneGo, St. Joseph, MI) were used for pathway analysis of DE gene between different samples. According to the P-value of each pathway, we chose the first 50 pathways of each gene set.

## Availability of supporting data

The data used in this study is available at the NCBI GEO database (http://www.ncbi.nlm.nih.gov/projects/geo, accession number GSE49247)

## Competing interests

The authors declare that they have no competing interests.

## Authors' contributions

RZ, LH, JY, JX JW and RL participated in the design of the experimental plan. RZ, LH, LW and MC take part in statistical analysis. WL performed wet-lab experiments. RZ drafted the manuscript, which was improved by JX and JW. All authors read and approved the final manuscript.

## Supplementary Material

Additional File 1 **Saturation curves of UMC1**. Number of expressed genes (blue curve) and correlation of expression (red curve) are plotted with sequencing depth. Only mRNAs are selected for further analysis. Click here for file

Additional File 2 **Pearson's correlation coefficient scatter plots between two euploid iPSCs, UMC1 and UMC6**. Click here for file

Additional File 3 **List of DE genes between each aneuploidy and euploid iPSCs**. DE genes by DEGseq between each aneuploidy and euploid iPSCs are list in the table with p-value<0.05, q-value <0.05 and fold change >=1.5. Click here for file

Additional File 4 **Clustered heatmap of GO enrichment analysis**. GO terms found in all four aneuploid cell lines are shown. The color intensities indicate enrichment score of each GO term. Click here for file

Additional File 5 **KEGG/GO terms of DE genes on chromosome 3 in trisomy 22 and on chromosome 10 in trisomy 8**. KEGG/GO terms found in DE genes chromosome 3 in trisomy 22 and on chromosome 10 in trisomy 8 with p-value<0.05 and counts >2. Click here for file

Additional File 6 **Pathway analysis using GeneGo Pathway tool**. Each number represents the amount of functional terms found in each functional group. Click here for file

## Declarations

Publication of this article was funded by grant (2011CB965200) from the National Basic Research Program (973 Program), the Ministry of Science and Technology of the People's Republic of China.

This article has been published as part of *BMC Genomics *Volume 14 Supplement 5, 2013: Twelfth International Conference on Bioinformatics (InCoB2013): Computational biology. The full contents of the supplement are available online at http://www.biomedcentral.com/bmcgenomics/supplements/14/S5.

## References

[B1] Lejeune J Turpin R Gautier M [Mongolism; a chromosomal disease (trisomy)] Bulletin de l'Academie nationale de medecine 1959 143 11-12 256 265 13662687

[B2] Hassold T Hunt P To err (meiotically) is human: the genesis of human aneuploidy Nature reviews Genetics 2001 2 4 280 291 10.1038/35066065 11283700

[B3] Simpson JL Causes of fetal wastage Clinical obstetrics and gynecology 2007 50 1 10 30 10.1097/GRF.0b013e31802f11f6 17304022

[B4] Mao R Zielke CL Zielke HR Pevsner J Global up-regulation of chromosome 21 gene expression in the developing Down syndrome brain Genomics 2003 81 5 457 467 10.1016/S0888-7543(03)00035-1 12706104

[B5] Saran NG Pletcher MT Natale JE Cheng Y Reeves RH Global disruption of the cerebellar transcriptome in a Down syndrome mouse model Human molecular genetics 2003 12 16 2013 2019 10.1093/hmg/ddg217 12913072

[B6] Kahlem P Sultan M Herwig R Steinfath M Balzereit D Eppens B Saran NG Pletcher MT South ST Stetten G Transcript level alterations reflect gene dosage effects across multiple tissues in a mouse model of down syndrome Genome research 2004 14 7 1258 1267 10.1101/gr.1951304 15231742 PMC442140

[B7] Lyle R Gehrig C Neergaard-Henrichsen C Deutsch S Antonarakis SE Gene expression from the aneuploid chromosome in a trisomy mouse model of down syndrome Genome research 2004 14 7 1268 1274 10.1101/gr.2090904 15231743 PMC442141

[B8] Mao R Wang X Spitznagel EL Jr Frelin LP Ting JC Ding H Kim JW Ruczinski I Downey TJ Pevsner J Primary and secondary transcriptional effects in the developing human Down syndrome brain and heart Genome biology 2005 6 13 R107 10.1186/gb-2005-6-13-r107 16420667 PMC1414106

[B9] Lockstone HE Harris LW Swatton JE Wayland MT Holland AJ Bahn S Gene expression profiling in the adult Down syndrome brain Genomics 2007 90 6 647 660 10.1016/j.ygeno.2007.08.005 17950572

[B10] Chou CY Liu LY Chen CY Tsai CH Hwa HL Chang LY Lin YS Hsieh FJ Gene expression variation increase in trisomy 21 tissues Mammalian genome: official journal of the International Mammalian Genome Society 2008 19 6 398 405 10.1007/s00335-008-9121-1 18594911

[B11] Slonim DK Koide K Johnson KL Tantravahi U Cowan JM Jarrah Z Bianchi DW Functional genomic analysis of amniotic fluid cell-free mRNA suggests that oxidative stress is significant in Down syndrome fetuses Proceedings of the National Academy of Sciences of the United States of America 2009 106 23 9425 9429 10.1073/pnas.0903909106 19474297 PMC2687148

[B12] Lavon N Narwani K Golan-Lev T Buehler N Hill D Benvenisty N Derivation of euploid human embryonic stem cells from aneuploid embryos Stem Cells 2008 26 7 1874 1882 10.1634/stemcells.2008-0156 18450823

[B13] Biancotti JC Narwani K Buehler N Mandefro B Golan-Lev T Yanuka O Clark A Hill D Benvenisty N Lavon N Human embryonic stem cells as models for aneuploid chromosomal syndromes Stem Cells 2010 28 9 1530 1540 10.1002/stem.483 20641042

[B14] Narwani K Biancotti JC Golan-Lev T Buehler N Hill D Shifman S Benvenisty N Lavon N Human embryonic stem cells from aneuploid blastocysts identified by pre-implantation genetic screening In vitro cellular & developmental biology Animal 2010 46 3-4 309 316 10.1007/s11626-010-9303-5 20224970 PMC2855810

[B15] Biancotti JC Narwani K Mandefro B Golan-Lev T Buehler N Hill D Svendsen CN Benvenisty N The in vitro survival of human monosomies and trisomies as embryonic stem cells Stem cell research 2012 9 3 218 224 10.1016/j.scr.2012.07.002 22892439

[B16] Takahashi K Tanabe K Ohnuki M Narita M Ichisaka T Tomoda K Yamanaka S Induction of pluripotent stem cells from adult human fibroblasts by defined factors Cell 2007 131 5 861 872 10.1016/j.cell.2007.11.019 18035408

[B17] Li W Wang X Fan W Zhao P Chan YC Chen S Zhang S Guo X Zhang Y Li Y Modeling abnormal early development with induced pluripotent stem cells from aneuploid syndromes Human molecular genetics 2012 21 1 32 45 10.1093/hmg/ddr435 21949351

[B18] Shi Y Kirwan P Smith J MacLean G Orkin SH Livesey FJ A human stem cell model of early Alzheimer's disease pathology in Down syndrome Science translational medicine 2012 4 124 124ra129 22344463 10.1126/scitranslmed.3003771 PMC4129935

[B19] FitzPatrick DR Ramsay J McGill NI Shade M Carothers AD Hastie ND Transcriptome analysis of human autosomal trisomy Human molecular genetics 2002 11 26 3249 3256 10.1093/hmg/11.26.3249 12471051

[B20] Nawata H Kashino G Tano K Daino K Shimada Y Kugoh H Oshimura M Watanabe M Dysregulation of gene expression in the artificial human trisomy cells of chromosome 8 associated with transformed cell phenotypes PloS one 2011 6 9 e25319 10.1371/journal.pone.0025319 21980425 PMC3183047

[B21] Bahn S Mimmack M Ryan M Caldwell MA Jauniaux E Starkey M Svendsen CN Emson P Neuronal target genes of the neuron-restrictive silencer factor in neurospheres derived from fetuses with Down's syndrome: a gene expression study Lancet 2002 359 9303 310 315 10.1016/S0140-6736(02)07497-4 11830198

[B22] Epstein CJ The consequences of chromosome imbalance American journal of medical genetics Supplement 1990 7 31 37 2149968 10.1002/ajmg.1320370706

[B23] FitzPatrick DR Transcriptional consequences of autosomal trisomy: primary gene dosage with complex downstream effects Trends in genetics: TIG 2005 21 5 249 253 10.1016/j.tig.2005.02.012 15851056

[B24] Costa V Angelini C D'Apice L Mutarelli M Casamassimi A Sommese L Gallo MA Aprile M Esposito R Leone L Massive-scale RNA-Seq analysis of non ribosomal transcriptome in human trisomy 21 PloS one 2011 6 4 e18493 10.1371/journal.pone.0018493 21533138 PMC3080369

[B25] Xiong Y Chen X Chen Z Wang X Shi S Zhang J He X RNA sequencing shows no dosage compensation of the active X-chromosome Nature genetics 2010 42 12 1043 1047 10.1038/ng.711 21102464

[B26] Marguerat S Bahler J RNA-seq: from technology to biology Cellular and molecular life sciences: CMLS 2010 67 4 569 579 10.1007/s00018-009-0180-6 19859660 PMC2809939

[B27] Allison DB Cui X Page GP Sabripour M Microarray data analysis: from disarray to consolidation and consensus Nature reviews Genetics 2006 7 1 55 65 10.1038/nrg1749 16369572

[B28] Marioni JC Mason CE Mane SM Stephens M Gilad Y RNA-seq: an assessment of technical reproducibility and comparison with gene expression arrays Genome research 2008 18 9 1509 1517 10.1101/gr.079558.108 18550803 PMC2527709

[B29] Dennis G Jr Sherman BT Hosack DA Yang J Gao W Lane HC Lempicki RA DAVID: Database for Annotation, Visualization, and Integrated Discovery Genome biology 2003 4 5 P3 10.1186/gb-2003-4-5-p3 12734009 PMC193660

[B30] Upender MB Habermann JK McShane LM Korn EL Barrett JC Difilippantonio MJ Ried T Chromosome transfer induced aneuploidy results in complex dysregulation of the cellular transcriptome in immortalized and cancer cells Cancer research 2004 64 19 6941 6949 10.1158/0008-5472.CAN-04-0474 15466185 PMC4772432

[B31] Bixby JL Harris WA Molecular mechanisms of axon growth and guidance Annual review of cell biology 1991 7 117 159 10.1146/annurev.cb.07.110191.001001 1687312

[B32] Dickinson RE Duncan WC The SLIT-ROBO pathway: a regulator of cell function with implications for the reproductive system Reproduction 2010 139 4 697 704 10.1530/REP-10-0017 20100881 PMC2971463

[B33] Andrews W Liapi A Plachez C Camurri L Zhang J Mori S Murakami F Parnavelas JG Sundaresan V Richards LJ Robo1 regulates the development of major axon tracts and interneuron migration in the forebrain Development 2006 133 11 2243 2252 10.1242/dev.02379 16690755

[B34] Ren T Anderson A Shen WB Huang H Plachez C Zhang J Mori S Kinsman SL Richards LJ Imaging, anatomical, and molecular analysis of callosal formation in the developing human fetal brain The anatomical record Part A, Discoveries in molecular, cellular, and evolutionary biology 2006 288 2 191 204 16411247 10.1002/ar.a.20282

[B35] Robinow M Haney N Chen H Sorauf T Van Dyke DL Babu VR Powell S Maliszewski W Guerin S Landers JW Secondary trisomy or mosaic "tetrasomy" 8p American journal of medical genetics 1989 32 3 320 324 10.1002/ajmg.1320320309 2729351

[B36] Markov D Ivanov S Popivanova P [Warkany syndrome associated with agenesis of the corpus callosum] Akusherstvo i ginekologiia 2007 46 2 48 50 17469453

[B37] Carter MT St Pierre SA Zackai EH Emanuel BS Boycott KM Phenotypic delineation of Emanuel syndrome (supernumerary derivative 22 syndrome): Clinical features of 63 individuals American journal of medical genetics Part A 2009 149A 8 1712 1721 10.1002/ajmg.a.32957 19606488 PMC2733334

[B38] Klein R Bidirectional modulation of synaptic functions by Eph/ephrin signaling Nature neuroscience 2009 12 1 15 20 10.1038/nn.2231 19029886

[B39] Balci S Gucer S Orhan D Karagoz T A well-documented trisomy 13 case presenting with a number of common and uncommon features of the syndrome The Turkish journal of pediatrics 2008 50 6 595 599 19227428

[B40] Chacon MR Fazzari P FAK: dynamic integration of guidance signals at the growth cone Cell adhesion & migration 2011 5 1 52 55 10.4161/cam.5.1.13681 20953136 PMC3038098

[B41] Niclou SP Jia L Raper JA Slit2 is a repellent for retinal ganglion cell axons The Journal of neuroscience: the official journal of the Society for Neuroscience 2000 20 13 4962 4974 10864954 10.1523/JNEUROSCI.20-13-04962.2000 PMC6772294

[B42] Feldheim DA Kim YI Bergemann AD Frisen J Barbacid M Flanagan JG Genetic analysis of ephrin-A2 and ephrin-A5 shows their requirement in multiple aspects of retinocollicular mapping Neuron 2000 25 3 563 574 10.1016/S0896-6273(00)81060-0 10774725

[B43] Woo S Rowan DJ Gomez TM Retinotopic mapping requires focal adhesion kinase-mediated regulation of growth cone adhesion The Journal of neuroscience: the official journal of the Society for Neuroscience 2009 29 44 13981 13991 10.1523/JNEUROSCI.4028-09.2009 19890008 PMC2796108

[B44] Lorke DE Winking H Histogenesis of the retina in murine trisomy 19 Brain research bulletin 1986 16 6 845 851 10.1016/0361-9230(86)90080-8 3756537

[B45] Chan A Lakshminrusimha S Heffner R Gonzalez-Fernandez F Histogenesis of retinal dysplasia in trisomy 13 Diagnostic pathology 2007 2 48 10.1186/1746-1596-2-48 18088410 PMC2244598

[B46] Guan CB Xu HT Jin M Yuan XB Poo MM Long-range Ca2+ signaling from growth cone to soma mediates reversal of neuronal migration induced by slit-2 Cell 2007 129 2 385 395 10.1016/j.cell.2007.01.051 17448996

[B47] Henley J Poo MM Guiding neuronal growth cones using Ca2+ signals Trends in cell biology 2004 14 6 320 330 10.1016/j.tcb.2004.04.006 15183189 PMC3115711

[B48] Moreland RS Cilea J Moreland S Calcium dependent regulation of vascular smooth muscle contraction Advances in experimental medicine and biology 1991 308 81 94 10.1007/978-1-4684-6015-5_7 1801589

[B49] O'Gorman CS Syme C Lang J Bradley TJ Wells GD Hamilton JK An evaluation of early cardiometabolic risk factors in children and adolescents with Turner syndrome Clinical endocrinology 2013 78 6 907 913 10.1111/cen.12079 23106295

[B50] Michael SK Surks HK Wang Y Zhu Y Blanton R Jamnongjit M Aronovitz M Baur W Ohtani K Wilkerson MK High blood pressure arising from a defect in vascular function Proceedings of the National Academy of Sciences of the United States of America 2008 105 18 6702 6707 10.1073/pnas.0802128105 18448676 PMC2373316

[B51] Hermes M Eichhoff G Garaschuk O Intracellular calcium signalling in Alzheimer's disease Journal of cellular and molecular medicine 2010 14 1-2 30 41 10.1111/j.1582-4934.2009.00976.x 19929945 PMC3837603

[B52] Boveri T Über mehrpolige Mitosen als Mittel zur analyse des zellkerns Verh d physmed Ges Würzburg N F 1902 35 67 9017867586

[B53] Duesberg P Rasnick D Li R Winters L Rausch C Hehlmann R How aneuploidy may cause cancer and genetic instability Anticancer research 1999 19 6A 4887 4906 10697602

[B54] Schoch C Kohlmann A Dugas M Kern W Schnittger S Haferlach T Impact of trisomy 8 on expression of genes located on chromosome 8 in different AML subgroups Genes, chromosomes & cancer 2006 45 12 1164 1168 10.1002/gcc.20380 17001623

[B55] Mertens F Sallerfors B Heim S Johansson B Kristoffersson U Malm C Mitelman F Trisomy 13 as a primary chromosome aberration in acute leukemia Cancer genetics and cytogenetics 1991 56 1 39 44 10.1016/0165-4608(91)90360-7 1747868

[B56] Virtaneva K Wright FA Tanner SM Yuan B Lemon WJ Caligiuri MA Bloomfield CD de La Chapelle A Krahe R Expression profiling reveals fundamental biological differences in acute myeloid leukemia with isolated trisomy 8 and normal cytogenetics Proceedings of the National Academy of Sciences of the United States of America 2001 98 3 1124 1129 10.1073/pnas.98.3.1124 11158605 PMC14719

[B57] Hasle H Olsen JH Nielsen J Hansen J Friedrich U Tommerup N Occurrence of cancer in women with Turner syndrome British journal of cancer 1996 73 9 1156 1159 10.1038/bjc.1996.222 8624281 PMC2074404

[B58] Schoemaker MJ Swerdlow AJ Higgins CD Wright AF Jacobs PA Cancer incidence in women with Turner syndrome in Great Britain: a national cohort study The lancet oncology 2008 9 3 239 246 10.1016/S1470-2045(08)70033-0 18282803

[B59] Farooque A Atapattu N Amarasena S Hogler W English MW Kirk JM An association of craniopharyngioma in Turner syndrome Pediatric blood & cancer 2013 60 6 E7 9 10.1002/pbc.24411 23255311

[B60] Cloonan N Forrest AR Kolle G Gardiner BB Faulkner GJ Brown MK Taylor DF Steptoe AL Wani S Bethel G Stem cell transcriptome profiling via massive-scale mRNA sequencing Nature methods 2008 5 7 613 619 10.1038/nmeth.1223 18516046

[B61] Lu X Shapiro JA Ting CT Li Y Li C Xu J Huang H Cheng YJ Greenberg AJ Li SH Genome-wide misexpression of X-linked versus autosomal genes associated with hybrid male sterility Genome research 2010 20 8 1097 1102 10.1101/gr.076620.108 20511493 PMC2909572

[B62] Wang L Feng Z Wang X Zhang X DEGseq: an R package for identifying differentially expressed genes from RNA-seq data Bioinformatics 2010 26 1 136 138 10.1093/bioinformatics/btp612 19855105

