# Supplementary figures and images for: Gene expression analysis of induced pluripotent stem cells from aneuploid chromosomal syndromes
Source: BMC Genomics. 2013 Oct 16;14(Suppl 5):S8. doi: 10.1186/1471-2164-14-S5-S8 (PMC3852284; doi:10.1186/1471-2164-14-S5-S8)

# Saturation curves

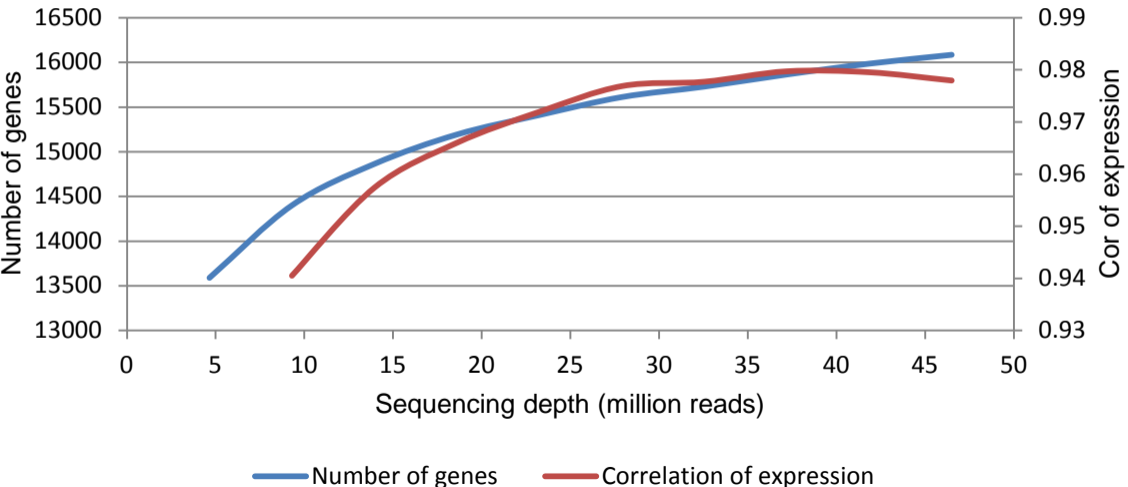

Supplement: Additional File 1 — Saturation curves of UMC1. Number of expressed genes (blue curve) and correlation of expression (red curve) are plotted with sequencing depth. Only mRNAs are selected for further analysis. [file 1471-2164-14-S5-S8-S1.pdf]

$R=0.967$

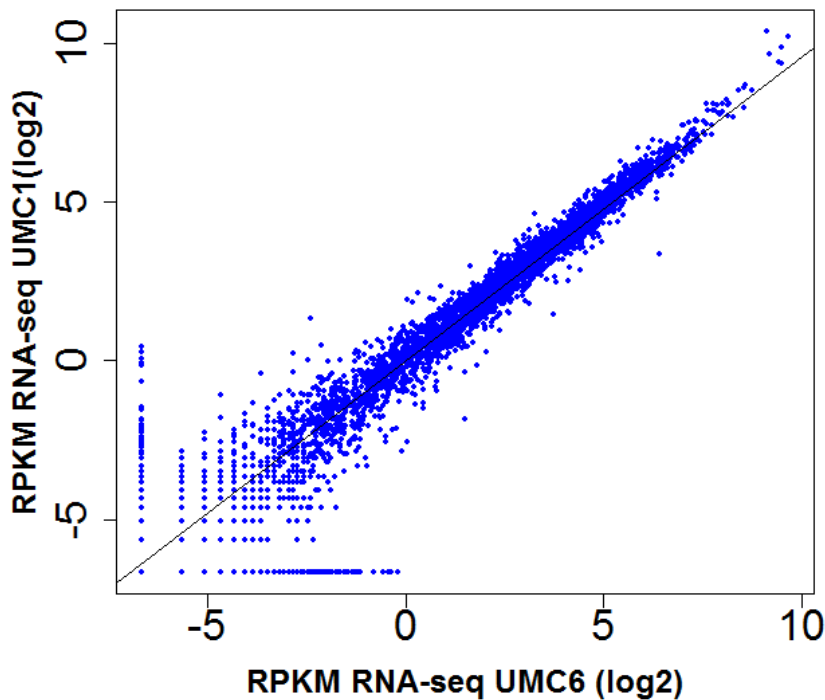

Supplement: Additional File 2 — Pearson's correlation coefficient scatter plots between two euploid iPSCs, UMC1 and UMC6. [file 1471-2164-14-S5-S8-S2.pdf]

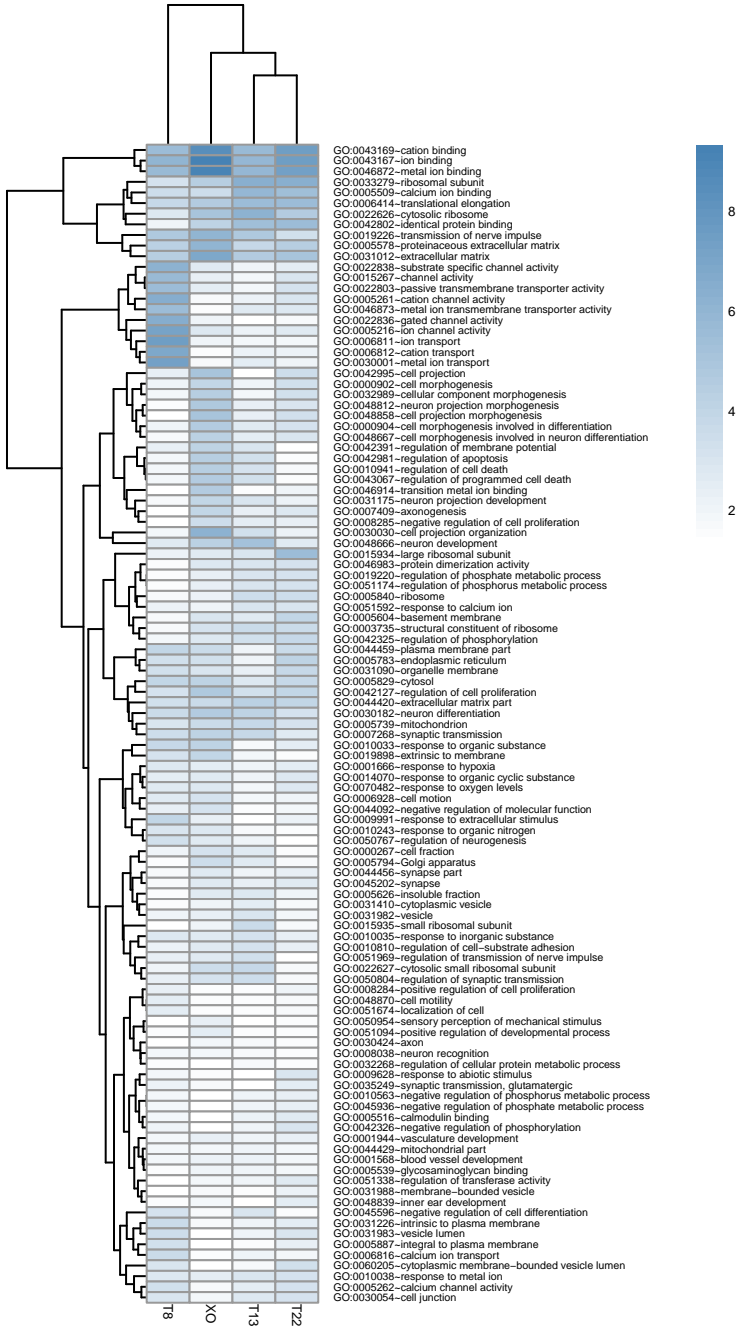

Supplement: Additional File 4 — Clustered heatmap of GO enrichment analysis. GO terms found in all four aneuploid cell lines are shown. The color intensities indicate enrichment score of each GO term. [file 1471-2164-14-S5-S8-S4.pdf]
