# Supplementary material for: Gene expression analysis of induced pluripotent stem cells from aneuploid chromosomal syndromes
Source: BMC Genomics. 2013 Oct 16;14(Suppl 5):S8. doi: 10.1186/1471-2164-14-S5-S8 (PMC3852284; doi:10.1186/1471-2164-14-S5-S8)
Supplement: Additional File 6 — Pathway analysis using GeneGo Pathway tool. Each number represents the amount of functional terms found in each functional group. [file 1471-2164-14-S5-S8-S6.doc]

| **GeneGo pathway groups** | **T8** | **T13** | **T22** | **XO** |
| --- | --- | --- | --- | --- |
| Apoptosis & Survival | 3 | 6 | - | 3 |
| Blood coagulation | 1 | 1 | 3 | - |
| Cell adhesion | 5 | 3 | 4 | 3 |
| Cytoskeleton remodeling | 7 | 6 | 6 | 6 |
| Development | 8 | 13 | 14 | 15 |
| G-protein signaling | 2 | 1 | 4 | 5 |
| Immune response | 15 | 9 | 6 | 9 |
| Signal transduction | 4 | 2 | 3 | 3 |
| Transcription | - | 2 | - | 1 |
| Translation | 1 | 1 | 2 | - |
